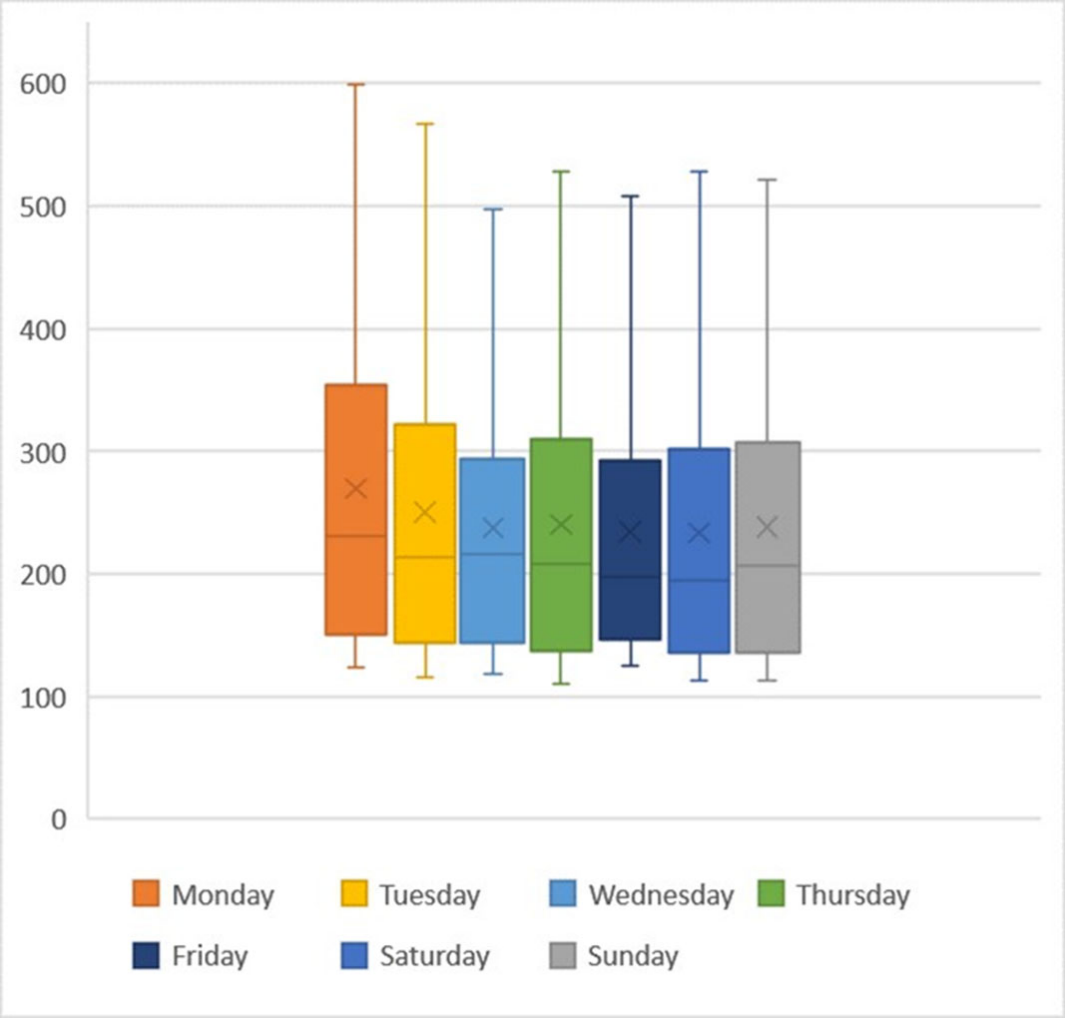# Blood Culture Utilization at Six Southeastern US Hospitals

**DOI:** 10.1017/ash.2021.64

**Published:** 2021-07-29

**Authors:** Bobby Warren, Rebekah Moehring, Michael Yarrington, Deverick Anderson, Christopher Polage

## Abstract

**Group Name:** Duke Center for Antimicrobial Stewardship and Infection Prevention

**Background:** Blood cultures are an essential diagnostic test, but over- and underutilization may cause harm. **Methods:** We analyzed blood culture utilization at 6 hospitals in the southeastern United States including 1 academic hospital (A) and 5 community hospitals (B–F) from May 2019 to April 2020. We measured blood culture utilization rate (BCUR) per 1,000 patient days and blood cultures per encounter. We counted blood cultures by laboratory accession number and measured utilization per 1,000 patient days and encounter. A likely contaminant was defined as 1 of 2 blood cultures collected in the same calendar day positive for a common skin commensal (CSC), as defined by the NHSN, and not identified from subsequent cultures. A likely pathogen was defined as a culture with a pathogen not on the CSC list or a CSC not meeting the contaminant definition. Hospital-level BCUR included samples for culture collected in the emergency department (ED) and inpatient areas divided by inpatient days. **Results:** The analysis included 117,897 blood cultures and 662,723 patient days with a median BCUR of 209.7 per hospital and median blood culture per encounter of 2 (Table 1). One community hospital (C) demonstrated a substantially higher BCUR than others. Cultures were frequently collected in the ED (54%; range, 36%–78%); most encounters with cultures in the ED were subsequently admitted to an inpatient unit (84%; range, 73%–89%). Higher BCURs were observed in intensive care and oncology units. The proportion of first blood cultures drawn after initiation of antibiotics was 6% (range, 3%–9%. Mondays had higher BCURs than other days of the week (Figure 1). The average BCUR by month was 176.1 (range, 164.3–181.4) with no seasonal patterns observed. Overall, 7.7% (range, 4.5%–9.1%) of blood cultures identified a likely pathogen and 2.1% (range, 1.3%–3.2%) identified a likely contaminant. The 3 hospitals with BCURs >200 also had contaminant rates >2% and >60% ED cultures. **Conclusions:** Blood culture utilization varied by hospital, unit, and day of the week. We observed higher rates of likely contaminants among hospitals with higher BCURs and ED culture rates. Comparisons may assist in identifying opportunities to optimize practice around blood-culture ordering and collection.

**Funding:** No

**Disclosures:** None

**Table 1.**
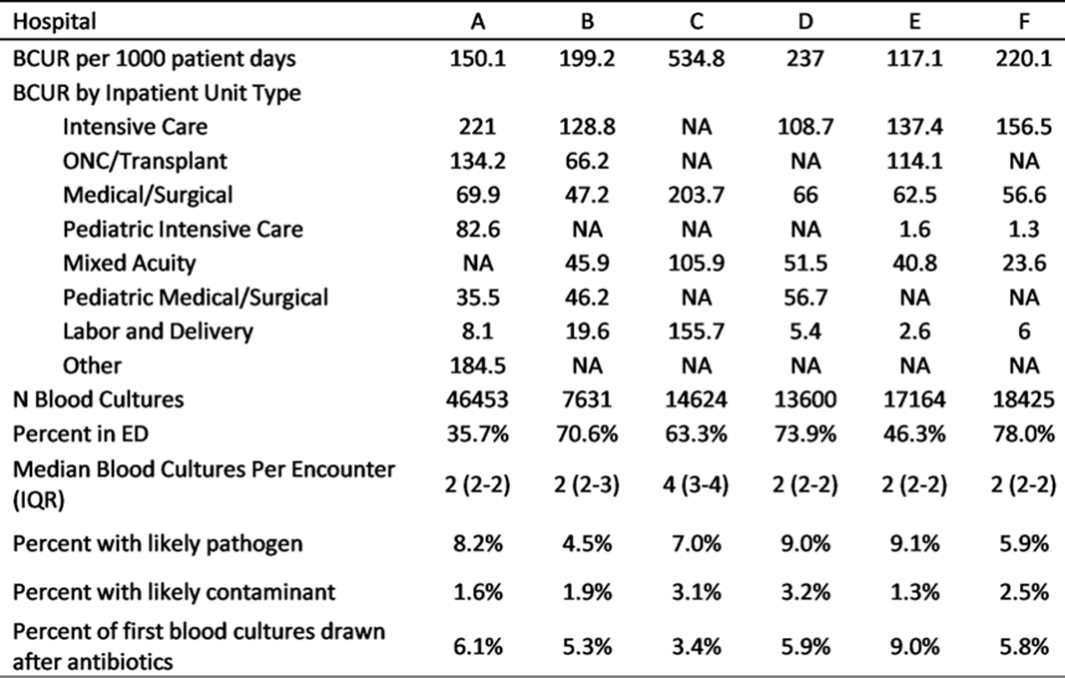

**Figure 1.**